# Microplasma-assisted green synthesis of glucose-stabilized silver nanoparticles: a dual-functional platform for SERS detection and synergistic reduction of binary dyes

**DOI:** 10.1039/d5ra09286h

**Published:** 2026-01-15

**Authors:** Pham The Tan, Truong Quang Giang, Tran Thu Trang, Vu Xuan Hoa, Luu Tuan Duong, Ngo Thi Lan, Nguyen Thi Luyen, Nguyen Van Hao

**Affiliations:** a Hung Yen University of Technology and Education Viet Tien Ward Hung Yen Province Vietnam; b TNU – University of Sciences Phan Dinh Phung Ward Thai Nguyen Province Vietnam haonv@tnus.edu.vn

## Abstract

A rapid and environmentally sustainable approach for synthesizing glucose-stabilized silver nanoparticles (G-AgNPs) was developed using an atmospheric-pressure microplasma process that completely eliminates the need for conventional chemical reductants and surfactants. The synergistic interaction between plasma-generated reactive species and glucose molecules enabled the one-step formation of uniformly dispersed AgNPs exhibiting dual morphologies—spherical (∼8 nm) and hexagonal (∼16 nm)—with distinct localized surface plasmon resonances (LSPR) centered at ∼403 nm. These nanostructures produced abundant electromagnetic “hot spots,” functioning as highly sensitive and reproducible SERS substrates capable of detecting Rhodamine 6G at concentrations as low as 10^−9^ M (enhancement factor = 8.31 × 10^7^, RSD = 4.85%, *n* = 9). Simultaneously, the G-AgNPs demonstrated excellent catalytic activity toward the NaBH_4_-assisted reduction of methylene blue (MB) and rhodamine B (RhB), following pseudo-first-order kinetics with rate constants of *k*_MB_ = 0.111 min^−1^ and *k*_RhB_ = 0.071 min^−1^ for single-dye systems, and *k*_MB_ = 0.085 min^−1^ and *k*_RhB_ = 0.068 min^−1^ for the binary mixture (*R*^2^ ≥ 0.97). The enhanced redox performance is consistent with a Langmuir–Hinshelwood-type surface-mediated mechanism, in which the glucose shell promotes electrostatic adsorption, mediates interfacial electron transfer, and enhances the colloidal stability of the AgNPs. By coupling plasmonic amplification with efficient catalytic reduction, the proposed microplasma-glucose strategy introduces a novel dual-functional nanoplatform for trace-level molecular detection and sustainable pollutant remediation.

## Abstract 2

A green, rapid method for synthesizing glucose-stabilized silver nanoparticles (G-AgNPs) using atmospheric-pressure microplasma is reported, eliminating conventional chemical reductants and surfactants.

## Graphical abstract

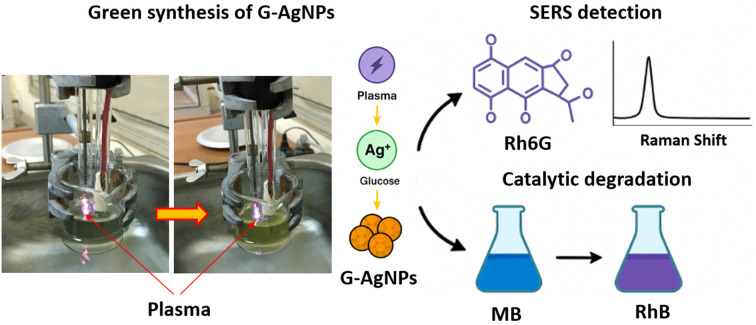

## 1. Introduction

Synthetic dyes such as methylene blue (MB), rhodamine B (RhB), and rhodamine 6G (Rh6G) are widely utilized in textile, paper, and leather manufacturing owing to their vibrant coloration, high photostability, and chemical robustness. However, their persistence, limited biodegradability, and toxicity pose significant environmental and health hazards, particularly to aquatic ecosystems.^1,2^ Residual dyes in wastewater reduce light penetration, suppress photosynthetic activity, and disturb the ecological balance.^3^ Conventional remediation methods—such as adsorption, coagulation, and biological degradation—are often inadequate for fully mineralizing these complex aromatic structures, thereby underscoring the urgent need for efficient, sustainable catalytic alternatives.^4^

Silver nanoparticles (AgNPs) have attracted extensive interest due to their unique physicochemical and plasmonic properties, which enable broad applications in catalysis, chemical sensing, and biomedicine.^5–7^ In particular, the localized surface plasmon resonance (LSPR) of AgNPs enhances light–matter interactions, leading to strong electromagnetic field localization at the nanoparticle surface and underpinning both surface-enhanced Raman scattering (SERS) and plasmon-assisted catalytic reactions.^8,9^ In SERS applications, AgNP-based substrates generate intense electromagnetic “hot spots” that can amplify Raman signals by several orders of magnitude, enabling ultratrace molecular detection down to the 10^−9^–10^−10^ M level,^10,11^ including in recently reported engineered plasmonic architectures.^12,13^ Beyond sensing, the strong plasmonic response of AgNPs also facilitates rapid interfacial electron transfer and provides abundant active surface sites, making them effective catalysts for the reduction of organic dyes in aqueous media.^8,9,14^

Despite their outstanding performance, conventional chemical synthesis of AgNPs typically relies on hazardous reducing or capping agents, such as NaBH_4_, hydrazine, or CTAB, which produce toxic by-products and limit biocompatibility.^15–17^ Consequently, environmentally sustainable synthetic strategies have been explored using benign precursors derived from plant extracts, amino acids, polysaccharides, or simple sugars.^18–21^ Among these, glucose acts as a dual-function reagent: its aldehyde group reduces Ag^+^ to metallic Ag^0^, while multiple hydroxyl moieties stabilize the nanoparticles and prevent agglomeration.^22–24^ Glucose-functionalized AgNPs exhibit excellent dispersibility in aqueous media and facilitate electrostatic or hydrogen-bonding interactions with dye molecules, making them particularly advantageous for catalytic and SERS applications.^22,24^

Atmospheric-pressure microplasma synthesis has recently emerged as a sustainable, high-efficiency alternative for the rapid fabrication of metal nanoparticles. Non-thermal microplasmas generate reactive species – such as solvated electrons (e^−^), hydroxyl radicals (˙OH), and hydrogen atoms (˙H) – that effectively reduce metal ions under ambient conditions without external chemical reductants.^25–28^ The plasma-liquid interface provides a localized environment for controlled nucleation and growth, resulting in phase-pure, monodisperse nanostructures.^26,28^ Numerous studies have demonstrated plasma-assisted synthesis of Au, Ag, and Cu nanoparticles with tunable morphologies and sizes achieved within minutes.^28–30^ When combined with biomolecular stabilizers, this technique yields ultra-clean nanomaterials exhibiting enhanced optical and catalytic functionalities. In particular, glucose-assisted microplasma synthesis offers simultaneous *in situ* reduction and surface passivation in a single-step, environmentally benign process.

The morphology and size-dependent characteristics of AgNPs play a critical role in determining their plasmonic and catalytic behaviors. While spherical nanoparticles typically display a single dipolar plasmon resonance, anisotropic morphologies, such as triangular or hexagonal plates, support multiple resonance modes, intensifying local electromagnetic fields and improving SERS sensitivity.^29–31^ Faceted hexagonal AgNPs, possessing sharp edges and corner sites, act as highly efficient electromagnetic hot spots,^32^ whereas smaller nanoparticles (∼5–10 nm) provide high surface-to-volume ratios favorable for catalytic redox reactions.^33,34^ Plasma-assisted approaches have been shown to produce Ag nanostructures with enhanced LSPR responses and catalytic reactivity compared with conventional wet-chemical methods.^35–38^ Notably, Yasin *et al.*^23^ reported fructose-stabilized AgNPs synthesized *via* microplasma, which exhibited rapid dye-degradation kinetics, although detailed SERS functionality was not explored.

Photoinduced morphological transformations of Ag nanoparticles have also been widely studied. A landmark report by Jin *et al.* demonstrated light-driven conversion of silver nanospheres into triangular nanoprisms *via* photon-assisted oxidative etching and facet-selective growth.^39^ In contrast, microplasma-driven synthesis relies on reductive plasma chemistry and radical-assisted pathways, enabling direct formation of anisotropic Ag nanostructures during synthesis without post-irradiation processing. This mechanistic distinction underscores the complementary nature of plasma-based approaches relative to photochemical shape control.

To clarify the scope of novelty and distinguish the present study from prior plasma-assisted, carbohydrate-mediated, and dual-function AgNP systems, a comparative summary of representative approaches is provided in Table 1. While individual elements such as plasma synthesis or glucose reduction have been reported previously, their mechanistic integration into a rapid, single-step process that yields dual-morphology AgNPs with combined catalytic and SERS functionality remains limited.

**Table 1:** Comparison of representative Ag nanoparticle synthesis strategies and the present plasma-glucose system in terms of synthesis conditions, morphology control, functional performance, and sustainability

Synthesis approach	Reductant/stabilizer	Energy input	Reaction time	Morphology control	SERS performance	Catalytic performance	Sustainability	References
Wet-chemical reduction	NaBH_4_/citrate	None	Seconds – minutes	Spherical	EF ∼10^5^–10^6^	Dye reduction	Uses strong chemical reductants	Creighton *et al.*^40^
Photoinduced shape conversion	Citrate	Visible light	Several hours	Triangular nanoprisms	Very high at sharp edges	Not central	Energy-intensive	Jin *et al.*^39^
Thermal chemical reduction	Glucose	Heating (60–90 °C)	2–6 h	Mostly spherical	Moderate SERS	Limited reports	Green but slow	Song *et al.*^41^
Solution plasma	None	Plasma discharge	5–30 min	Size-controlled spheres	SERS substrates	Photocatalysis	No chemical reductant	Yoshida *et al.*^35^
Microplasma-liquid	Fructose	Plasma discharge	10–20 min	Small spherical NPs	Not explored	Dye degradation	Green stabilizer	Yasin *et al.*^23^
Chemical multistep synthesis	Organic ligands	Chemical	Hours	Edged-satellite hybrids	High SERS, biosensing	Capture/Antibacterial	Complex synthesis	Wu *et al.*^12^
Atmospheric microplasma-glucose	Glucose (reductant + stabilizer + mediator)	Plasma discharge	≤ 10 min	Dual (spherical + hexagonal)	EF = 8.31 × 10^7^; LOD = 10^−9^ M	MB/RhB reduction	Single-step, green	This study

In this study, we report a one-step, green synthesis of glucose-stabilized silver nanoparticles (G-AgNPs) using atmospheric microplasma and systematically investigate their structural, optical, catalytic, and SERS properties. The NaBH_4_ – mediated degradation of MB and RhB-both individually and as a binary dye mixture, was analyzed in relation to particle morphology and plasmonic response, while SERS detection of Rh6G was employed to elucidate the interplay among electromagnetic enhancement, surface adsorption, and charge-transfer processes. The collective results demonstrate that the G-AgNPs function as robust, biocompatible nanocatalysts with dual catalytic and sensing capabilities, offering promising potential for sustainable environmental remediation and pollutant monitoring.

## 2. Materials and methods

### 2.1. Chemicals

Silver nitrate (AgNO_3_), d-glucose, methylene blue (MB), rhodamine B (RhB), rhodamine 6G (Rh6G), and sodium borohydride (NaBH_4_) were obtained from commercial suppliers and used as received. Deionized water (≥18 MΩ cm) was used throughout. High-purity argon (99.99%) was employed during microplasma synthesis.

### 2.2. Synthesis of glucose-stabilized silver nanoparticles (G-AgNPs)

Glucose-stabilized AgNPs (G-AgNPs) were synthesized using an atmospheric-pressure microplasma system operating under ambient conditions. An aqueous AgNO_3_ solution containing glucose was subjected to microplasma irradiation for a fixed duration, during which plasma-generated reactive species induced rapid reduction of Ag^+^ ions and simultaneous surface stabilization. Detailed configuration of the plasma reactor and operating parameters are provided in SI (Section S1).

### 2.3. Characterization

The morphology and size distribution of the synthesized G-AgNPs were examined by transmission electron microscopy (TEM). Phase structure and crystallinity were analyzed by X-ray diffraction (XRD). Optical properties and localized surface plasmon resonance behavior were analyzed using UV-visible spectroscopy. The surface functional groups and molecular interactions responsible for nanoparticle stabilization were investigated by Fourier-transform infrared spectroscopy. Detailed instrumental parameters and experimental conditions are provided in the SI (Section S2).

### 2.4. SERS measurements

Raman spectroscopy with a 532 nm excitation laser was employed for SERS measurements. SERS performance of G-AgNPs was evaluated using rhodamine 6G (Rh6G) as a probe molecule over a wide concentration range. Raman spectra were recorded after mixing Rh6G solutions with G-AgNP colloids. The SERS enhancement factor (EF) was calculated by comparing SERS and normal Raman signals using a standard approach. Full experimental parameters and data processing procedures are described in the SI (Section S3).

### 2.5. Catalytic degradation experiments

The catalytic activity of G-AgNPs was assessed through NaBH_4_-mediated reduction of methylene blue (MB) and rhodamine B (RhB) in aqueous solution. Under excess NaBH_4_ conditions, the reactions followed pseudo-first-order kinetics, and apparent rate constants were extracted from UV-visible absorption measurements. Detailed experimental protocols and kinetic analysis procedures are provided in the SI (Section S4).

## 3. Results and discussion

### 3.1. Structural characterization of G-AgNPs

**Fig. 1:**
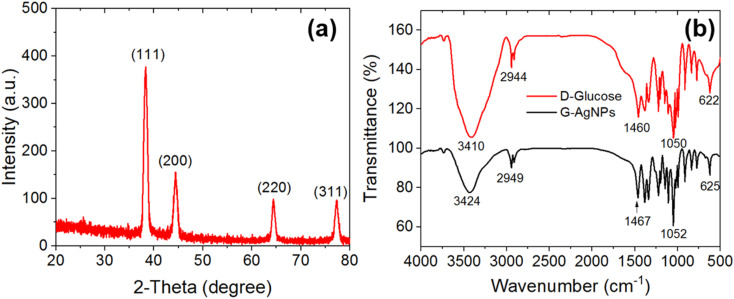
(a) XRD pattern of G-AgNPs; (b) FTIR spectra of d-glucose and G-AgNPs synthesized *via* plasma–liquid interaction.

The crystalline structure of the glucose-stabilized silver nanoparticles (G-AgNPs) was characterized using X-ray diffraction (XRD). As shown in Fig. 1a, four distinct diffraction peaks located at 2*θ* = 38.39°, 44.63°, 64.71°, and 77.40° can be indexed to the (111), (200), (220), and (311) planes of face-centered cubic (fcc) metallic silver (JCPDS card No. 04-0783). The absence of additional peaks related to silver oxide or other impurities confirms the formation of phase-pure, crystalline Ag nanoparticles. This diffraction pattern is consistent with AgNPs synthesized *via* plasma-assisted or green chemical reduction methods.^23,42^ The average crystallite size, calculated from the full width at half maximum (FWHM) of the (111) reflection using the Scherrer equation:1
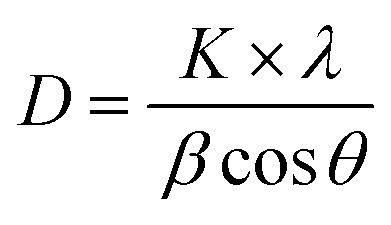
where *K* = 0.9, *λ* = 0.154056 nm, *β* is FWHM in radians, and *θ* is the Bragg angle, was estimated to be approximately 8.6 nm, which aligns well with the particle dimensions obtained from TEM analysis. This agreement confirms the nanoscale crystalline nature of the synthesized G-AgNPs and the effective size control achieved through plasma-liquid reduction.

To investigate glucose-induced surface functionalization of AgNPs, Fourier-transform infrared (FTIR) spectroscopy was performed, as illustrated in Fig. 1b. The FTIR spectrum of pristine d-glucose exhibits a broad O–H stretching band at ∼3410 cm^−1^. In the G-AgNPs spectrum, this band shifts slightly to ∼3424 cm^−1^ with noticeable broadening and reduced intensity, indicating a modification of the hydrogen-bonding environment upon interaction with the Ag nanoparticle surface. This small blue-shift (Δ*ν* ≈ +14 cm^−1^) suggests partial involvement of hydroxyl groups in surface coordination rather than the formation of strong covalent bonds.^43^

The C–H stretching vibration remains nearly unchanged (2944 → 2949 cm^−1^), indicating preservation of the glucose backbone after microplasma treatment. Meanwhile, the C–O stretching band shows a slight shift from ∼1050 to ∼1052 cm^−1^ with reduced intensity, implying weak Ag–O interactions involving vicinal diol groups. These quantitative spectral changes support the role of glucose as a surface-passivating ligand that stabilizes Ag nanoparticles through hydrogen bonding and weak coordination, while preserving sufficient surface accessibility for catalytic and SERS processes.^22^

Taken together with the FTIR, TEM, and UV-vis results, these observations suggest that d-glucose plays a dual role in the microplasma synthesis process, acting as a mild reductant and as a surface-passivating ligand. The presence of a hydroxyl-rich organic shell contributes to suppressing nanoparticle agglomeration and maintaining colloidal stability, which provides a consistent structural basis for the optical, catalytic, and SERS properties discussed in the following sections.

### 3.2. UV-vis spectroscopy and formation mechanism of G-AgNPs *via* microplasma

**Fig. 2:**
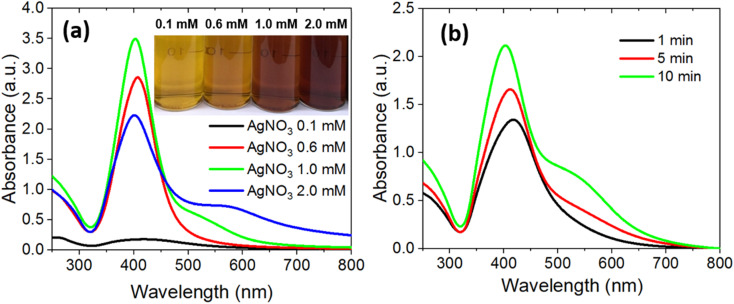
UV-vis absorption spectra of G-AgNPs synthesized *via* microplasma–liquid interaction at (a) varying AgNO_3_ concentrations for 10 min and (b) varying plasma exposure times at 2.0 mM AgNO_3_.

The optical properties and growth evolution of G-AgNPs were examined by UV-vis spectroscopy to monitor localized surface plasmon resonance (LSPR) formation. As shown in Fig. 2a, all samples exhibit a prominent plasmonic band around 403 nm, typical of spherical AgNPs.^44^ Increasing the AgNO_3_ concentration from 0.1 to 2.0 mM leads to a gradual increase in absorbance intensity and a visible color shift from pale yellow to dark brown, indicating higher nanoparticle yield and enhanced plasmonic coupling. At the highest precursor concentration (2.0 mM), the appearance of a broad shoulder near 580 nm suggests the formation of larger or anisotropic species, such as hexagonal nanoplates, with multiple plasmon modes.

Time-dependent UV-vis spectra recorded at a fixed AgNO_3_ concentration (2.0 mM) under varying plasma exposure times (Fig. 2b) reveal that nanoparticle nucleation occurs rapidly within 1 minute of discharge. The LSPR intensity increases rapidly up to 5 minutes, followed by a slight red-shift and peak broadening at 10 minutes, likely due to Ostwald ripening or partial aggregation under prolonged irradiation.^45^ These trends demonstrate the high efficiency of the plasma-liquid reduction process, enabling complete AgNP formation within minutes, significantly faster than conventional chemical routes.

The microplasma-assisted synthesis of G-AgNPs proceeds through a combined electrochemical and radical-mediated reduction mechanism driven by reactive species at the plasma–liquid interface. Immediately after discharge initiation, the solution changes from colorless to yellow (Fig. 2a inset), indicating rapid nucleation of metallic Ag. The overall formation mechanism can be described in the following steps:

(i) Anodic water oxidation generates O_2_ and protons.

2 2H_2_O − 4e^−^ → O_2_↑ + 4H^+^,

(ii) Cathodic reduction of Ag^+^ ions to metallic Ag^0^.

3 Ag^+^ + e^−^ → Ag^0^

4 Accompanied by hydrogen evolution 2H_2_O + 2e^−^ → H_2_↑ + 2OH^−^

(iii) Migration of Ag^+^/H^+^ toward the plasma–liquid interface.

(iv) Nucleation and growth of Ag clusters through atomic coalescence, and

(v) Surface passivation by glucose, where hydroxyl coordination stabilizes newly formed nanoparticles and suppresses secondary aggregation.

This pathway enables controlled nanoparticle formation, with glucose capping enhancing colloidal stability, as confirmed by FTIR analysis (Fig. 1b). Compared with previously reported mechanisms, the plasma-induced reduction and anisotropic evolution observed here are fundamentally distinct from classical photoinduced processes.^39^ Jin *et al.* demonstrated that the conversion of Ag nanospheres into triangular nanoprisms requires photon-assisted oxidative etching and facet-selective growth mediated by halides or surface adsorbates. In contrast, our microplasma-glucose system forms both spherical and hexagonal Ag nanostructures directly during synthesis through plasma-generated solvated electrons and radical species (e_aq_^−^, H˙, OH˙), without the need for post-irradiation reshaping. Furthermore, existing plasma-assisted syntheses predominantly yield spherical or monodisperse Ag nanoparticles and do not report dual spherical–hexagonal morphologies or glucose-mediated anisotropic growth. This comparison highlights that the anisotropic evolution described here proceeds through a distinct plasma-driven, reductive radical pathway, highlighting the distinctiveness of the present synthetic approach.

Optical emission spectroscopy (OES) was employed to qualitatively probe the reactive microplasma environment during G-AgNP synthesis. Characteristic emissions corresponding to OH radicals, atomic hydrogen, oxygen species, and excited argon confirm efficient plasma-induced water dissociation and generation of reducing species (e^−^, OH˙, H˙) responsible for the rapid reduction of Ag^+^ to Ag^0^. Detailed emission assignments and plasma reaction pathways are provided in the SI (Section S5).

### 3.3. Morphological analysis of G-AgNPs

**Fig. 3:**
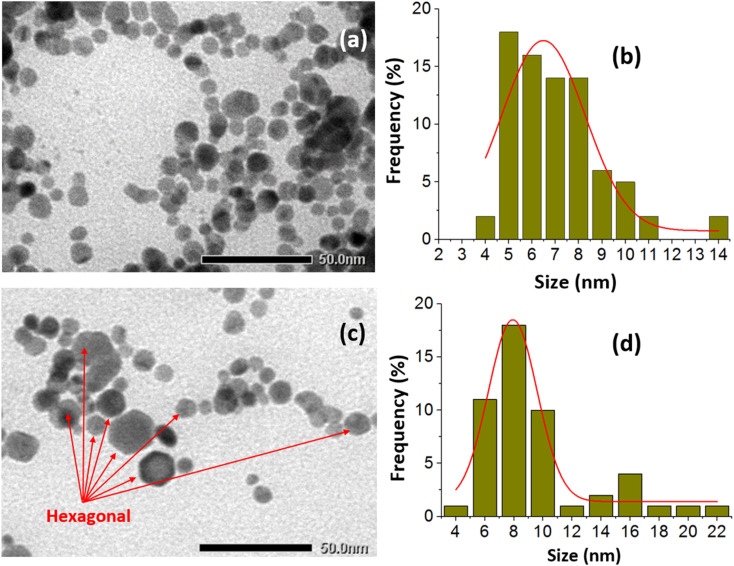
TEM images of G-AgNPs synthesized at (a) 0.6 mM AgNO_3_ and (c) 2.0 mM AgNO_3_ for 10 min, with corresponding size distribution histograms (b) and (d).

Transmission electron microscopy (TEM) was employed to elucidate the morphology, size distribution, and growth behavior of the glucose-stabilized silver nanoparticles. As depicted in Fig. 3a, G-AgNPs prepared at 0.6 mM are predominantly spherical and monodisperse, exhibiting uniform contrast and well-defined boundaries. The corresponding particle size histogram (Fig. 3b), based on measurements of over 200 nanoparticles using ImageJ, shows an average diameter of 8.1 ± 1.2 nm, confirming nanoscale uniformity and minimal aggregation.

Increasing the AgNO_3_ concentration to 2.0 mM induces clear morphological diversification (Fig. 3c). The TEM images reveal a bimodal population comprising both small (∼8 nm) spherical nanoparticles and larger anisotropic structures (∼16 nm), including hexagonal and pentagonal nanoplates. The size distribution histogram (Fig. 3d) exhibits two distinct peaks, confirming the coexistence of isotropic and anisotropic growth. The appearance of these faceted nanostructures is consistent with the additional ∼580 nm absorption shoulder in the UV-vis spectrum (Fig. 2a), attributed to multipolar plasmon modes of anisotropic Ag geometries.^46^

The transition from uniform spheres to faceted nanoplates can be rationalized by concentration-dependent nucleation kinetics and growth anisotropy during the microplasma-driven reduction process. At low Ag^+^ concentrations, rapid nucleation and restricted atomic diffusion favor isotropic particle formation. In contrast, higher precursor concentrations increase the local supersaturation and promote oriented attachment and selective facet growth along low-energy crystallographic planes. Plasma-generated electrons and radicals further accelerate reduction rate, creating non-equilibrium conditions that facilitate anisotropic development. Additionally, adsorbed glucose molecules act as facet-selective capping agents, preferentially binding to Ag(111) and Ag(100) planes and stabilizing high-energy edges, thereby inhibiting uncontrolled aggregation.

This synergistic interplay between plasma-assisted reduction and glucose-mediated surface passivation produces well-dispersed, shape-controlled nanostructures enriched with edge and corner sites, key contributors to localized electromagnetic “hot spots” that enhance SERS activity. Consequently, the glucose-assisted microplasma route not only enables precise morphological control but also tunes plasmonic coupling and catalytic performance, establishing a versatile platform for multifunctional nanomaterial design.

### 3.4. Evaluation of SERS sensitivity using rhodamine 6G as a probe molecule

The surface-enhanced Raman scattering (SERS) performance of G-AgNPs was systematically evaluated using Rhodamine 6G (Rh6G) as a probe molecule. Rh6G was selected owing to its well-defined vibrational signatures and environmental relevance as a model organic pollutant.^47^ SERS substrates were prepared by drop-casting G-AgNP suspensions synthesized at two AgNO_3_ concentrations (0.5 and 2.0 mM) onto glass slides, enabling a comparative assessment of how nanoparticle morphology and surface coverage affect enhancement performance.

**Fig. 4:**
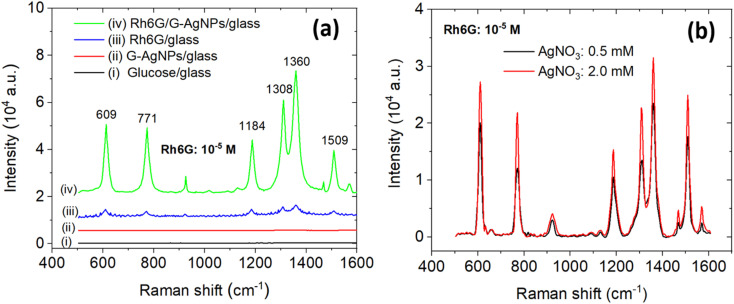
(a) Comparative Raman spectra of (i) glucose-coated glass substrate, (ii) bare G-AgNPs on glass, (iii) 10^−5^ M Rh6G on bare glass, and (iv) 10^−5^ M Rh6G on G-AgNP-modified glass substrate (synthesized from 2.0 mM AgNO_3_), (b) SERS spectra of 10^−5^ M Rh6G on G-AgNP substrates synthesized from 0.5 mM (black) and 2.0 mM (red) AgNO_3_ precursor concentrations.

As shown in Fig. 4a, the Raman spectra of four representative samples – (i) glucose/glass, (ii) G-AgNPs/glass, (iii) Rh6G/glass, and (iv) Rh6G/G-AgNPs/glass (10^−5^ M Rh6G) – illustrate the pronounced SERS effect. Bare glass and G-AgNPs/glass exhibit only weak background features, confirming negligible intrinsic Raman activity. In contrast, the Rh6G/G-AgNPs substrate displays intense, well-resolved peaks at 609, 771, 1184, 1308, 1360, and 1509 cm^−1^, corresponding to in-plane C–C–C ring deformation, out-of-plane and in-plane C–H bending, and aromatic C–C stretching modes of the xanthene structure, respectively.^48–50^ The dramatic signal amplification and sharp vibrational features confirm efficient electromagnetic enhancement on the G-AgNPs surface.

The primary source of signal enhancement originates from the localized surface plasmon resonance (LSPR) of AgNPs, which generates intense nanoscale electromagnetic (EM) fields, commonly referred to as “hotspots” – at particle junctions, tips, and edges.^51^ A secondary contribution from the chemical (charge-transfer) mechanism further intensifies the Raman response, particularly for the aromatic stretching modes near 1360 and 1509 cm^−1^, arising from photo-induced electron exchange between the Ag Fermi level and the π* orbitals of Rh6G.^52,53^

Fig. 4b clearly demonstrates that the SERS intensity of Rh6G strongly depends on the AgNO_3_ concentration employed during the microplasma synthesis. Increasing the precursor concentration from 0.5 to 2.0 mM leads to a substantial enhancement of the Raman signal, with the characteristic band at 1360 cm^−1^ exhibiting an approximately 2.5-fold increase in intensity. This behavior is consistent with the morphological evolution observed in TEM images, where the sample prepared at 0.5 mM predominantly consists of small spherical nanoparticles, whereas the 2.0 mM sample exhibits a higher fraction of polygonal nanostructures. The coexistence of particles with different sizes and shapes is expected to statistically increase the number of interparticle junctions, thereby promoting stronger electromagnetic field localization.

Importantly, the enhanced SERS response observed here cannot be attributed solely to the morphology of individual nanoparticles, but rather to the heterogeneous assembly of spherical and polygonal Ag nanostructures. Such mixed configurations increase both the diversity and spatial distribution of interparticle junctions, which act as electromagnetic hot spots. This heterogeneity, together with enhanced interparticle coupling effects, is widely recognized as a dominant factor governing SERS enhancement in realistic nanoparticle-based substrates.

This structure–property relationship is further supported by the UV-vis spectrum (Fig. 3a), where the appearance of a secondary absorption shoulder around 580 nm indicates multipolar plasmon coupling among adjacent anisotropic AgNPs.^54^ Additionally, the slight red-shift (∼5 cm^−1^) and broadening of the 1360 cm^−1^ Raman band for the 2.0 mM sample suggest stronger interparticle electromagnetic coupling within a more complex plasmonic environment. The glucose capping layer, as evidenced by FTIR (Fig. 1b), stabilizes the nanoparticles while preserving adequate surface accessibility for analyte adsorption. Unlike traditional surfactant-based stabilizers, this organic capping minimizes charge-screening effects and facilitates charge transfer across the metal-molecule interface.^55^

Taken together, these results demonstrate that microplasma-derived G-AgNPs provide morphology-tunable and highly reproducible SERS performance, where the Ag precursor concentration serves as a key parameter for controlling nanoparticle geometry, surface density, and hotspot distribution. The combined effects of plasmonic coupling and glucose-mediated stabilization enable sensitive, uniform, and environmentally benign detection of Rh6G at ultra-trace level, demonstrating the potential of this approach for practical environmental sensing applications.

**Fig. 5:**
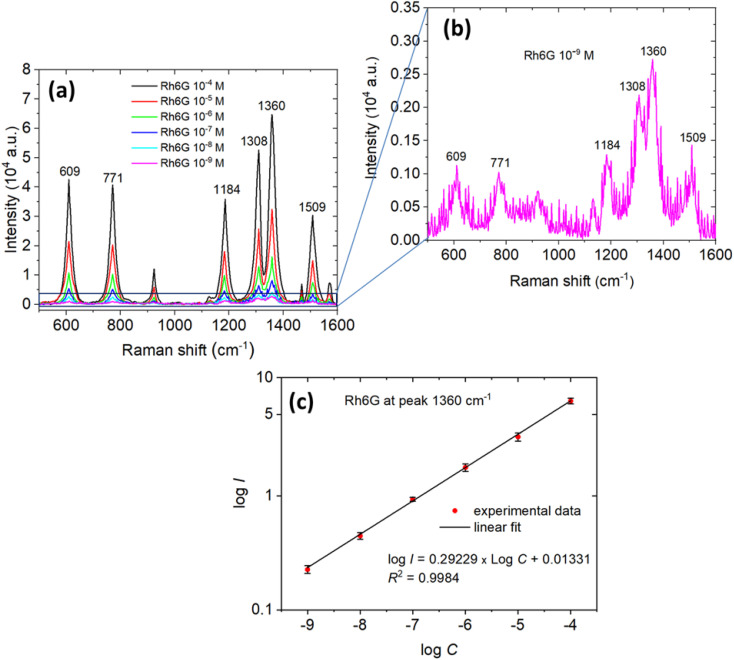
(a) Concentration-dependent SERS spectra of Rh6G on G-AgNP-modified glass substrates synthesized from 2.0 mM AgNO_3_, covering a range from 10^−4^ M to 10^−9^ M, (b) magnified view of the SERS spectrum at 10^−9^ M Rh6G, highlighting the retention of key vibrational bands (*e.g.*, 1360 cm^−1^) indicative of the substrate's ultralow limit of detection. (c) Logarithmic calibration curve plotting the intensity of the 1360 cm^−1^ peak *versus* the logarithm of Rh6G concentration, showing a linear relationship (*R*^2^ = 0.98) and confirming quantitative sensitivity across the tested concentration range.

The detection performance of the optimized G-AgNPs substrate was evaluated using Rh6G over a broad concentration range (10^−4^–10^−9^ M), as presented in Fig. 5a. As the analyte concentration decreased, the characteristic Raman bands of Rh6G at 609, 771, 1184, 1308, 1360, and 1509 cm^−1^ diminished in intensity while retaining consistent peak positions and spectral profiles. This spectral invariance in vibrational signatures confirms the structural stability and uniform field distribution of the G-AgNPs substrate, indicating that plasmonic enhancement remains highly effective even at trace-level molecular levels.

Remarkably, well-defined Raman signals with high signal-to-noise ratios remain detectable even at 10^−9^ M (Fig. 5b), particularly for the aromatic C–C stretching mode near 1360 cm^−1^. This detection limit is several orders of magnitude lower than that achievable by conventional Raman spectroscopy for fluorescent dyes such as Rh6G,^7,54^ evidencing the ultrasensitive performance of the microplasma-synthesized G-AgNPs.

To assess the quantitative reproducibility of the SERS response, the logarithmic relationship between Raman intensity (I) at 1360 cm^−1^ and analyte concentration (C) was plotted (Fig. 5c). The resulting calibration curve exhibits excellent linearity (*R*^2^ = 0.9984) over five orders of magnitude, reflecting uniform hotspot distribution and homogeneous molecular adsorption across the substrate. The slope of approximately 0.85 suggests sub-monolayer adsorption consistent with a Langmuir-type adsorption equilibrium, supported by the glucose coating, which provides sterically accessible and electrostatically favorable binding sites. The hydrophilic hydroxyl groups of glucose enhance dye-nanoparticle affinity *via* hydrogen bonding, while simultaneously preventing nanoparticle aggregation, thereby ensuring high repeatability and spectral uniformity.^56^

**Fig. 6:**
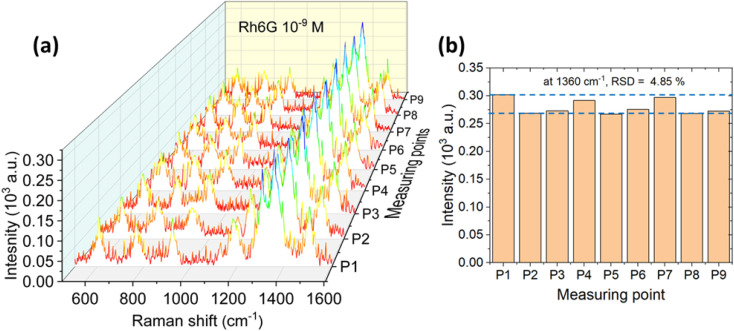
(a) SERS spectra of 10^−9^ M Rhodamine 6G (R6G) collected from 9 randomly selected spots on the G-AgNP-modified glass substrate synthesized from 2.0 mM AgNO_3_, (b) histogram of the intensity distribution for the 1360 cm^−1^ peak, showing a relative standard deviation (RSD) of 4.85%, calculated from the 9 measurements, underscoring the reproducibility of the SERS enhancement.

Minor deviations from linearity at higher concentrations likely originate from localized molecular aggregation or slight background variations; however, the overall response demonstrates outstanding reproducibility and quantitative reliability of the SERS signal. Compared with conventional chemical or solvent-based routes, the microplasma synthesis offers markedly improved detection limit, reproducibility, and inter-batch consistency (Fig. 6). The *in situ* generation of reactive plasma species (e^−^, ˙H, ˙OH) enables precise control over nucleation kinetics, facet-selective growth, and interparticle spacing, leading to a well-ordered plasmonic landscape with optimized charge-transfer efficiency.

These synergistic advantages establish the G-AgNPs substrate as a robust, eco-friendly, and highly reproducible SERS platform capable of detecting ultra-trace pollutants and molecular intermediates *in situ*. The dual functionality of the microplasma-glucose system, combining plasmonic amplification with catalytic activity, underscores its potential for real-time environmental monitoring and advanced remediation applications.^4,57^

Fig. 6a presents the SERS spectra of Rhodamine 6G (Rh6G, 10^−9^ M) collected from nine randomly selected regions on the G-AgNPs substrate synthesized at 2.0 mM AgNO_3_. All spectra consistently exhibit the characteristic Raman modes of R6G at 609, 771, 1184, 1308, 1360, and 1509 cm^−1^, with the aromatic C–C stretching vibration at 1360 cm^−1^ employed as a representative marker. The nearly identical peak positions and signal intensities across all sampling locations confirm the excellent spatial homogeneity and plasmonic uniformity of the G-AgNPs surface, both prerequisites for quantitative and reproducible SERS detection.

The reproducible detection of Rh6G down to 10^−9^ M (Fig. 5b or 6) highlights the robust signal amplification capability of the G-AgNPs substrate. Rather than reiterating spectral features, this performance reflects the effective coupling between the bimodal nanoparticle morphology and the plasmonic response discussed above, which collectively generates a high density of electromagnetic hot spots responsible for enhancement factors on the order of 10^6^–10^8^.^58,59^ The close similarity among SERS spectra acquired from multiple surface locations (Fig. 6a) confirms excellent spatial uniformity and signal stability, ensuring reliable measurements even at ultralow analyte concentrations. These results demonstrate that the high SERS sensitivity originates not only from plasmonic resonance but also from uniform hotspot distribution enabled by the plasma–glucose synthesis route.

As shown in Fig. 6b, statistical evaluation of the 1360 cm^−1^ band intensity across multiple acquisition points yields a relative standard deviation (RSD) of 4.85%, indicating highly reproducible and spatially uniform SERS responses. This exceptional uniformity arises from the microplasma-driven nucleation process, in which reactive species such as solvated electrons, hydroxyl radicals, and hydrogen atoms (Fig. S4) regulate Ag nucleation and growth in a spatially homogeneous manner.^60^ In contrast, conventional chemical reduction routes often produce broader particle-size distributions and higher RSD values (10–20%) due to uncontrolled reaction kinetics and localized aggregation.^61–64^

The low RSD obtained here surpasses most previously reported green-synthesized Ag-based SERS substrates, where variations in morphology and surface coverage commonly introduce significant spectral fluctuations.^65,66^ The microplasma-glucose synergy provides a self-regulated reaction environment: plasma-generated reducing species enable rapid, surfactant-free formation of metallic Ag, while glucose molecules coordinate to nanoparticle surfaces, maintaining dispersion and preventing aggregation without adding insulating organic layers. At higher precursor concentrations (2.0 mM AgNO_3_), the preferential growth of anisotropic Ag nanostructures enhances near-field coupling and hotspot density, thereby boosting overall SERS intensity. Collectively, these results demonstrate that microplasma-assisted glucose synthesis yields a highly uniform, reproducible, and chemically clean SERS substrate, representing an important step toward reliable, green-fabricated plasmonic materials for practical sensing and environmental analysis.

To quantify the plasmonic enhancement, the SERS enhancement factor (EF) was calculated for the characteristic Raman modes of Rh6G using the standard relation:
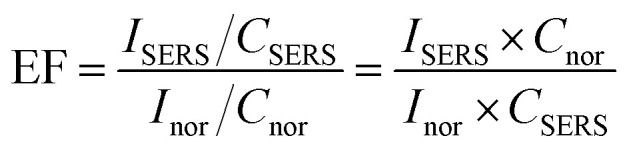
where *I*_SERS_ and *I*_nor_ denote the integrated Raman intensities obtained from SERS and normal Raman spectra, respectively, and *C*_SERS_ and *C*_nor_ represent the corresponding analyte concentrations. In this work, *C*_nor_ = 10^−2^ M was chosen to obtain a measurable Raman signal despite fluorescence interference, while *C*_SERS_ = 10^−6^ and 10^−9^ M were used for the G-AgNPs prepared with 2.0 mM AgNO_3_.

Raman spectra were baseline-corrected *via* Lorentzian fitting, and the intensities were averaged over nine independent measurements (*n* = 9), yielding a relative standard deviation of 4.85% (Fig. 6b). Under identical experimental conditions (*λ* = 532 nm, 1 mW, 10 s integration), the normal Raman intensity of Rh6G at 1360 cm^−1^ was 328 a.u.

The calculated enhancement factors (EFs) reveal a pronounced concentration-dependent SERS response of the G-AgNPs substrate, with EF values on the order of 10^4^–10^5^ at 10^−6^ M and reaching ∼10^6^ at 10^−9^ M (Table S2). This nonlinear increase in EF with decreasing analyte concentration suggests a transition from multilayer adsorption to a submonolayer regime, where molecule-metal charge-transfer interactions become increasingly significant within localized plasmonic hot spots. Such behavior correlates well with the bimodal nanoparticle morphology observed in TEM images (Fig. 3c and d), in which coexisting spherical and hexagonal Ag nanostructures promote heterogeneous interparticle coupling and multipolar plasmon resonances. The combined electromagnetic enhancement from LSPR (*λ* ≈ 403 nm) and chemical enhancement *via* charge-transfer interactions with the Rh6G π-system accounts for the superior SERS performance, exceeding that of conventional citrate-reduced AgNPs reported in the literature.^57,67^

As summarized in Table 2, the G-AgNPs substrate exhibits an enhancement factor (EF) of up to 8.31 × 10^7^ with a detection limit (LOD) of 10^−9^ M for Rh6G, placing it among the high-performance Ag-based SERS platforms reported to date. The achieved EF surpasses that of conventional citrate-derived AgNPs^65^ and rivals advanced engineered SERS platforms such as AgNP assemblies on Si nanopillars (EF = 10^6^–10^8^, 10^−10^ M),^68^ two-dimensional Ag nanowire networks (EF = 8 × 10^10^),^69^ and laser-deposited Ag nanodroplets (EF ≈ 8 × 10^8^ at <10^−9^ M).^70^ Other Ag-based systems such as random Ag aggregates (EF ≈ 10^6^ at 10^−7^ M),^71^ Ag@SiO_2_ (EF = 1.5 × 10^7^ at 10^−11^ M),^72^ and truncated Ag nanotriangles (EF = 2.3 × 10^7^ at 10^−8^ M),^48^ also exhibit strong enhancement but generally suffer from limited reproducibility and structural instability. Likewise, polymer-thiol-stabilized Ag films (EF ≈ 10^5^–10^6^)^73^ and three-dimensional Ag “flower-like” nanostructures (LOD = 10^−9^ M)^74^ show good uniformity yet moderate enhancement.

**Table 2:** Comparison of enhancement factors (EF) and limits of detection (LOD) for G-AgNPs-based SERS substrates detecting Rh6G

LOD (M)	EF (at LOD or equivalent)	Publication year	Remarks	References
10^−10^	10^6^–10^8^	2005	Self-assembled Ag NPs on silicon nanotips, high surface area for near-field amplification	68
Not specified	8 × 10^10^	2013	Two-dimensional netlike Ag nanowires, interparticle junctions and network structures	69
<10^−9^	∼8 × 10^8^	2016	Laser-deposited drop-shaped Ag nanostructures on silicon, dense hotspots from erosion torches	70
10^−7^	∼10^6^	2008	Silver aggregates, interparticle coupling	71
10^−11^	1.5 × 10^7^	2021	Ag@SiO_2_ nanospheres, improved stability and ordered arrangement	72
10^−8^	2.3 × 10^7^	2016	Truncated nanotriangular Ag NPs, shape-enhanced LSPR.	48
Not specified	∼10^5^–10^6^	2018	Thiol-immobilized polymer-capped Ag NPs on Si(100), high reproducibility	73
10^−9^	Not specified	2025	3D Ag nanoflowers with nano-gaps, salt-enhanced sensitivity	74
10^−9^	1.12 × 10^7^	2019	Tunable Ag NP arrays on nanocones, uniform gaps *via* hot embossing	61
10^−9^	∼10^6^–10^7^	2021	Ag-decorated Si nanowires, optimized for dye discrimination	75
10^−10^	1.92 × 10^6^	2022	Ag NPs on silicon pyramids, uniform deposition and anti-aggregation	66
10^−9^	Not specified	2019	Ag-decorated horizontal Si nanowires, moderate enhancement with careful design	76
10^−9^	8.31 × 10^7^ (at 1360 cm^−1^)	2025	Glucose-stabilized, microplasma-synthesized, bimodal morphology	This work

Among green-synthesized Ag nanomaterials, the G-AgNPs substrate exhibits an exceptionally high EF achieved *via* a single-step atmospheric microplasma-glucose process, which provides precise morphological control together with intrinsic biocompatibility. Although its EF is lower than that of highly engineered systems such as Ag nanowire meshes (EF = 8 × 10^10^),^69^ the combination of EF = 8.31 × 10^7^ and LOD = 10^−9^ M demonstrates strong practical competitiveness. Compared with tunable Ag arrays on Si nanocones (EF = 1.12 × 10^7^ at 10^−9^ M),^61^ Ag-decorated Si nanowires (EF ≈ 10^6^–10^7^),^75^ Ag-coated Si pyramids (EF = 1.92 × 10^6^ at 10^−10^ M),^66^ or Ag nanowire films,^76^ the G-AgNPs exhibit superior enhancement stability due to their dense and uniform hotspot distribution achieved without corrosive etchants or high-energy plasma processing.

Overall, the performance metrics summarized in Table 2 confirm that the G-AgNPs substrate (EF = 8.31 × 10^7^, LOD = 10^−9^ M) ranks among the most efficient green-prepared Ag-based SERS systems reported so far. Its unique combination of morphological tunability, high reproducibility (RSD = 4.85%), and intrinsic catalytic activity underscores its potential as a universal platform for ultrasensitive molecular sensing and sustainable environmental remediation – bridging the gap between plasmonic detection and green nanotechnology.

### 3.5. Catalytic performance of G-AgNPs

The catalytic activity of G-AgNPs was systematically investigated through the reduction of two representative organic dyes – methylene blue (MB) and rhodamine B (RhB), using sodium borohydride (NaBH_4_) as an electron donor. These dyes were selected due to their distinct structural and electronic characteristics: MB is a cationic phenothiazine dye, whereas RhB is a zwitterionic xanthene derivative whose charge state varies with pH. Their contrasting molecular features provide an effective platform for assessing redox selectivity, interfacial charge-transfer efficiency, and adsorption behavior on plasmonic catalysts.^5,77^

**Fig. 7:**
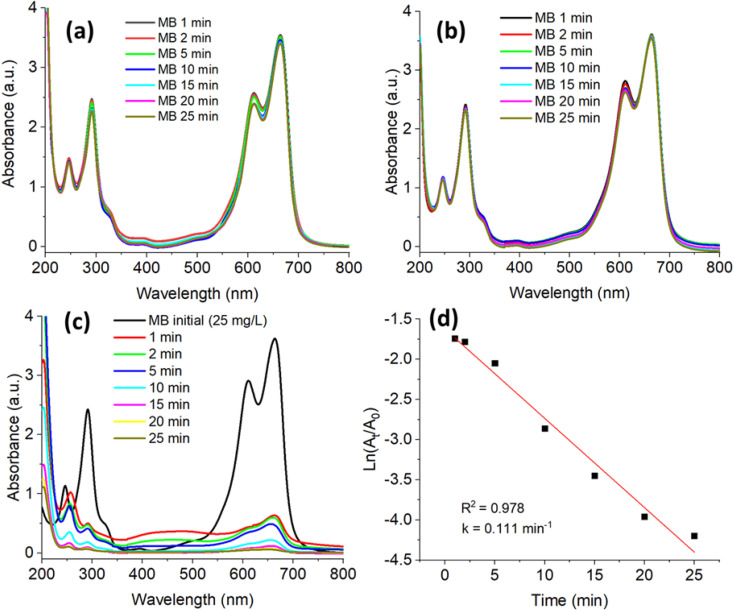
UV-vis spectra of MB degradation (a) by glucose alone, (b) by NaBH_4_ alone, (c) by NaBH_4_ in the presence of G-AgNPs and (d) first-order kinetics plotted for G-AgNPs.

**Fig. 8:**
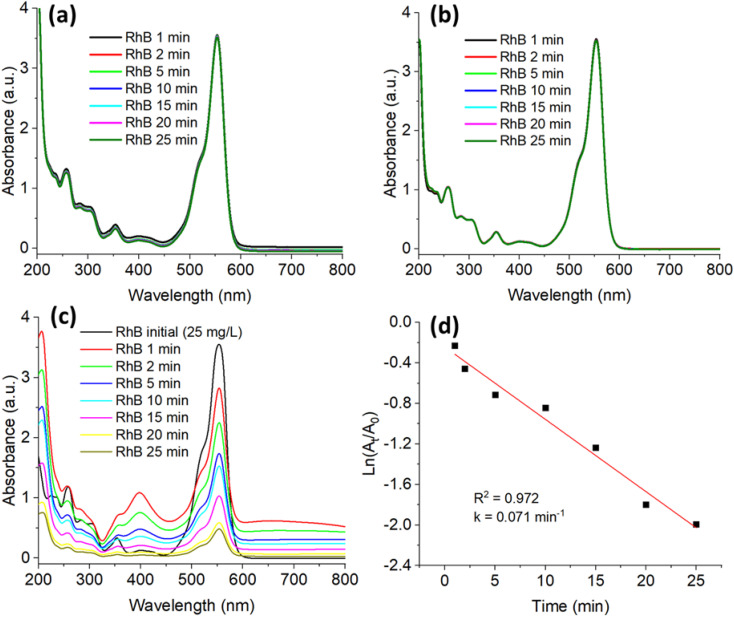
UV-vis spectra of RhB degradation (a) by glucose alone, (b) by NaBH_4_ alone, (c) by NaBH_4_ in the presence of G-AgNPs and (d) first-order kinetics plotted for G-AgNPs.

**Fig. 9:**
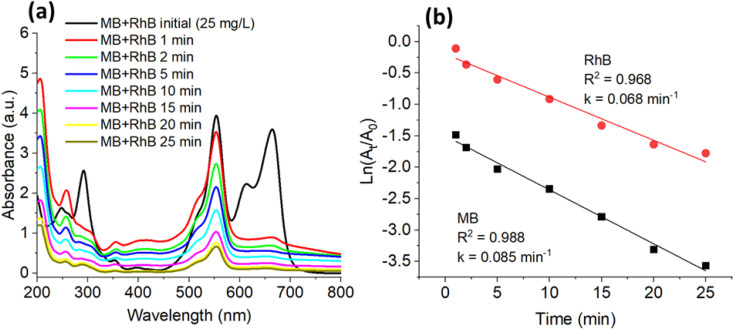
UV-vis spectra of MB and RhB degradation mixture (a) by NaBH_4_ in the presence of G-AgNPs and (b) first-order kinetics plotted for G-AgNPs.

**Fig. 10:**
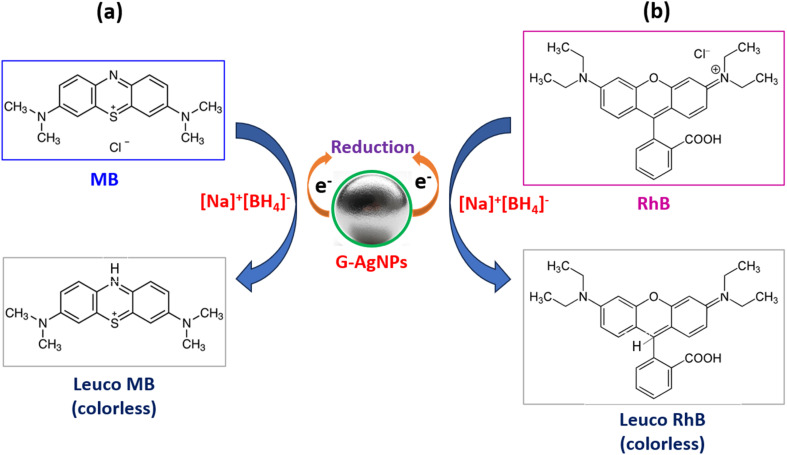
Schematic diagram of the mechanism of the catalytic reduction of MB (a) and RhB (b) by NaBH_4_ and G-AgNPs samples.

Fig. 7–10 present the time-dependent UV-vis absorption spectra, kinetic analyses, and the proposed reduction mechanism. The G-AgNPs exhibit rapid catalytic response and efficient electron mediation, evident from the accelerated decay of the characteristic absorption bands and excellent structural stability retained over multiple reaction cycles. In comparison with conventionally synthesized or biogenically derived AgNPs, the microplasma-generated G-AgNPs demonstrate superior turnover frequency and durability, highlighting the advantages of this hybrid synthesis route in producing catalytically active and environmentally benign plasmonic nanostructures.

Fig. 7 shows the UV-vis absorption spectra of MB under three conditions: (a) glucose only, (b) NaBH_4_ only, and (c) NaBH_4_ in the presence of G-AgNPs. In the first two case, the dominant absorption band of MB centered at 664 nm remains unchanged, confirming that neither glucose nor NaBH_4_ alone can effectively reduce MB at room temperature. In contrast, upon introducing G-AgNPs, the 664 nm peak decreases rapidly, and the solution becomes nearly colorless within 25 min, demonstrating the nanoparticles' strong catalytic activity.

The pronounced decline of the 664 nm band evidences a plasmon-assisted electron transfer process, in which G-AgNPs mediate charge flow from BH_4_^−^ donors to MB acceptors. The reaction is consistent with a Langmuir–Hinshelwood mechanism, involving co-adsorption of reactants on the Ag^0^ surface and subsequent hydride transfer that reduces the chromophoric –C=N^+^- moiety, disrupting the π–π conjugation and resulting in dye decolorization.^5,77^

The kinetic profile (Fig. 7d) follows a pseudo-first-order model, as indicated by the linear relationship between ln*(A*_*t*_*/A*_o_) and time, yielding a correlation coefficient of *R*^2^ = 0.978 and an apparent rate constant *k* = 0.111 ± 0.007 min^−1^. This value reflects a fast and stable reduction process under mild conditions. Although the rate constant is somewhat lower than those reported for AgNPs derived from *Arctium lappa* (*k* = 0.364 min^−1^)^18^ and d-fructose-capped AgNPs (*k* = 0.165 min^−1^),^23^ the discrepancy primarily arises from the higher MB concentration employed in this study (25 mg L^−1^*versus* ∼16 mg L^−1^) and differences in nanoparticle size, surface area, and capping-layer thickness, all of which influence the interfacial electron-transfer rate.

Despite the moderate rate constant, the G-AgNPs exhibit remarkable catalytic efficiency and long-term operational stability, attributable to their organic–inorganic hybrid structure. The hydroxyl-rich glucose shell enhances electrostatic adsorption of cationic MB molecules while preventing nanoparticle aggregation, thereby maintaining colloidal integrity throughout the reaction. Meanwhile, the metallic Ag^0^ core provides an efficient conductive pathway for electron transfer from BH_4_^−^ to MB, supporting sustained catalytic turnover.^78^ Furthermore, the microplasma-assisted synthesis yields nanoparticles with a narrow size distribution and controlled surface oxidation, ensuring reproducible catalytic performance across successive cycles. Collectively, these features establish G-AgNPs as a robust, recyclable, and eco-compatible plasmonic nanocatalyst for the efficient degradation of organic contaminants in aqueous systems.

To further support the proposed surface-mediated catalytic pathway, zeta potential measurements were performed (Fig. S3). The pristine G-AgNPs exhibit a moderately negative surface charge (−10.2 mV), attributable to glucose-derived surface functionalities. Upon interaction with methylene blue and NaBH_4_, distinct changes in zeta potential are observed, indicating adsorption of both dye molecules and borohydride species on the AgNP surface. These surface charge variations provide experimental support for a Langmuir–Hinshelwood-type mechanism involving co-adsorption and interfacial electron transfer, rather than a purely homogeneous reduction process.

Fig. 8 presents the time-resolved UV-vis absorption spectra of rhodamine B (RhB) recorded under three different experimental conditions: (a) glucose only, (b) NaBH_4_ only, and (c) NaBH_4_ in the presence of G-AgNPs. In the control systems (a) and (b), the principal RhB absorption band at 554 nm remains essentially unchanged after 25 min, confirming that neither glucose nor NaBH_4_ alone can induce RhB reduction at room temperature. In contrast, upon the addition of G-AgNPs, the 554 nm peak decreases rapidly and nearly disappears within 25 min, accompanied by a distinct color change from pink to colorless. This observation clearly indicates that the metallic Ag^0^ surface acts as an active electron-transfer interface, efficiently mediating the reduction of RhB without the need for photoirradiation.

Kinetic evaluation (Fig. 8d) demonstrates that the reaction follows a pseudo-first-order rate law, as evidenced by the linear dependence of ln(*A*_*t*_*/A*_o_) on time with an excellent correlation (*R*^2^ = 0.972). The apparent rate constant was determined to be *k* = 0.071 ± 0.005 min^−1^, which is lower than that obtained for MB reduction (*k* = 0.111 min^−1^). This difference reflects the inherent structural and electronic disparities between the two dyes. Specifically, RhB possesses a xanthene chromophore with bulky ethyl substituents, which limits adsorption on the Ag surface and weakens π-metal coupling, thereby reducing electron-transfer efficiency. The relatively slower kinetics are consistent with literature trends, where biogenic AgNPs synthesized using Trigonella foenum-graecum extract exhibited *k* ≈ 0.20 min^−1^,^19^ and poly-extract-mediated AgNPs showed comparable values (∼0.19–0.22 min^−1^).^79^ By contrast, a CeO_2_–NaBH_4_ catalytic system yielded a higher rate constant (*k* = 0.171 min^−1^) owing to the Ce^3+^/Ce^4+^ redox shuttle, which continuously regenerates active electrons.^80^

Despite its slightly lower apparent rate constant, the G-AgNPs catalyst exhibits excellent stability and reproducibility under ambient, light-free conditions and at relatively high RhB concentrations (25 mg L^−1^). The hydroxyl-rich glucose shell is proposed to facilitate electrostatic attraction and hydrogen-bonding interactions with cationic RhB molecules, thereby maintaining colloidal dispersion and suppressing nanoparticle coalescence during catalysis.

The catalytic reduction behavior is consistent with a Langmuir–Hinshelwood (L–H)-type surface-mediated mechanism, in which both BH_4_^−^ species and dye molecules are likely adsorbed on the metallic Ag^0^ surface prior to electron transfer. In this framework, electrons originating from BH_4_^−^ are transferred through the Ag surface to the adsorbed RhB molecules, promoting reduction of the chromophoric groups and gradual disruption of the extended π-conjugated system, ultimately leading to colorless leuco-rhodamine derivatives.

In addition, the plasmonic character of the Ag nanoparticles may contribute to interfacial charge polarization at the metal-solution interface, creating localized electromagnetic fields that transiently increase surface electron density and facilitate electron-transfer kinetics. Such plasmon-assisted effects have been widely reported to enhance reduction rates in noble-metal nanoparticle systems compared with non-plasmonic catalysts. The combined contributions of glucose stabilization, efficient surface adsorption, and plasmonic activation collectively account for the high catalytic selectivity and robustness of G-AgNPs toward RhB degradation.

Fig. 9 illustrates the catalytic degradation of a binary dye mixture comprising MB and RhB in the presence of NaBH_4_, catalyzed by the G-AgNPs system. The UV-vis spectra (Fig. 9a) display a progressive attenuation of the characteristic absorption peaks at 664 nm (MB) and 554 nm (RhB), accompanied by a gradual decolorization within approximately 40 min. This behavior indicates that the G-AgNPs effectively promote the simultaneous and complete reduction of both dyes under ambient, dark conditions.

Kinetic fitting (Fig. 9b) reveals that the degradation of both dyes follows a pseudo-first-order rate law, with apparent rate constants of *k*_MB_ = 0.085 min^−1^ (*R*^2^ = 0.988) and *k*_RhB_ = 0.068 min^−1^ (*R*^2^ = 0.968). The consistently higher rate constant for MB supports a sequential-synergistic degradation pattern characteristic of binary dye systems, whereby MB is preferentially reduced before RhB. This behaviour arises from differences in molecular structure and electronic properties: MB, a cationic phenothiazine dye, exhibits stronger electrostatic attraction to the hydroxyl-rich glucose shell, enhancing adsorption and interfacial charge transfer. In contrast, the zwitterionic RhB molecule experiences steric hindrance and charge delocalization, which reduces its surface affinity and retard electron transfer from the Ag^0^ core.

These results align with previous reports in multicomponent catalytic systems. Nagajyothi *et al.*^80^ observed that CeO_2_ derived from Ce-MOF preferentially reduced MB (*k*_MB_ > *k*_RhB_) in the presence of NaBH_4_, while Veisi *et al.* (2021)^81^ observed a similar trend for Au@biguanidine-KIT-5 in a ternary MB-RhB-MO system. Likewise, Yu *et al.* (2020)^82^ attributed the selective reduction of MB on cellulose-AgNP composites to strong electrostatic interactions between cationic MB and surface hydroxyl groups. Quantitatively, the *k*_MB_ = 0.085 min^−1^ obtained in this study is comparable to values reported for Ag/SiO_2_ catalysts (*k* ≈ 0.02 min^−1^)^83^ and notably higher than the rate for RhB reduction over Cestrum nocturnum-derived AgNPs.^79^ These comparisons confirm that the glucose layer not only stabilizes the Ag nanoparticles but also plays an active role in modulating adsorption geometry and electron-transfer kinetics.

The observed sequential-synergistic degradation mechanism can be rationalized by differences in molecular size, charge polarity, and adsorption energy, which govern competitive surface coverage and interfacial reactivity. Similar trends have been reported for modified SrTiO_3_ catalysts,^84^ CeO_2_/Co_3_O_4_/Ag/Ag_3_PO_4_ nanocomposites,^85^ and Ca_4_Fe_9_O_17_/biochar hybrids,^86^ where cooperative electron mediation between coexisting dyes enhances the overall catalytic rate through inter-dye charge transfer and suppresses recombination losses.

In the present system, the initial adsorption and rapid reduction of MB likely enhance local electron density and optical transparency, thereby promoting subsequent plasmon-assisted electron relay to RhB molecules adsorbed on active sites. This plasmonic-molecular synergy enables efficient dual-dye degradation without external irradiation, highlighting the advantage of the microplasma-glucose synthesis route in creating biocompatible, sustainable, and highly active Ag-based nanocatalysts for multifunctional applications in environmental remediation and SERS sensing.

Fig. 10 schematically illustrates the proposed catalytic pathway for the reduction of MB and RhB over G-AgNPs. In aqueous solution, NaBH_4_ dissociates to generate BH_4_^−^ ions, which are expected to adsorb on the Ag^0^ surface and act as electron donors. Concurrently, dye molecules are adsorbed *via* electrostatic and hydrogen-bonding interactions with the glucose-functionalized surface. Interfacial electron transfer from BH_4_^−^ to the surface-bound dyes induces stepwise reduction of electron-deficient moieties, including the iminium (–C=N^+^–) center in MB and the conjugated xanthene framework in RhB, resulting in chromophore bleaching and solution decolorization.^69,85^

Overall, the catalytic transformation can be rationalized within a Langmuir–Hinshelwood-type framework, where co-adsorption of reactants on the nanoparticle surface governs reaction kinetics. The glucose capping layer plays a multifunctional role by stabilizing Ag nanoparticles against aggregation, promoting adsorption of cationic dyes through hydroxyl-mediated interactions, and enabling efficient interfacial electron transfer, as commonly reported for carbohydrate-stabilized plasmonic nanocatalysts.^23^ While direct spectroscopic evidence of surface intermediates is not available in the present study, the kinetic behavior, adsorption-dependent trends, and literature precedents collectively support this mechanistic interpretation.

This cooperative interplay between the conductive Ag core and the organic glucose shell enables efficient surface reactions while maintaining catalyst durability and recyclability. The proposed mechanism highlights how the microplasma-glucose approach integrates plasmonic functionality with green surface chemistry, where plasmon-induced charge polarization and hot-carrier-assisted processes may further facilitate interfacial redox reactions, consistent with previous reports on noble-metal plasmonic catalysis.^86^

While plasma-assisted Ag nanoparticle synthesis, carbohydrate-mediated reduction, and dual-function SERS/catalytic systems have each been reported separately in the literature, the present work distinguishes itself by integrating these elements into a single-step, atmospheric microplasma-glucose platform. In this system, plasma-generated electrons drive rapid nucleation, glucose simultaneously serves as a green reductant, facet-directing stabilizer, and adsorption mediator, and the resulting statistically prevalent hexagonal Ag nanostructures provide enhanced plasmonic hot spots and interfacial electron-transfer pathways. This integrated mechanistic framework, rather than any individual component alone, underpins the observed dual functionality and sustainability of the G-AgNP system.

The present study is primarily intended to establish the fundamental dual-functional behavior of glucose-stabilized Ag nanoparticles synthesized *via* atmospheric microplasma, with emphasis on single-cycle SERS sensitivity and catalytic reduction efficiency. While the obtained results clearly demonstrate strong plasmonic enhancement and rapid surface-mediated catalysis, further investigations are required to fully assess the long-term practical applicability of this system. Future work will therefore focus on (i) evaluating catalytic durability over multiple reuse cycles (≥5 cycles), (ii) examining SERS signal retention and reproducibility under repeated measurements, and (iii) assessing colloidal and functional stability during extended storage periods (1–4 weeks) under ambient conditions. These systematic studies will enable a more comprehensive evaluation of the robustness, reusability, and shelf-life of G-AgNPs, which are critical parameters for their deployment in real-world sensing and environmental remediation applications.

## 4. Conclusion

This study demonstrates that atmospheric-pressure microplasma synthesis provides a controllable and environmentally benign route for producing glucose-stabilized Ag nanoparticles with bimodal morphology and pronounced plasmonic activity. The G-AgNPs exhibit dual functionality: highly sensitive SERS detection of Rh6G (LOD = 10^−9^ M, EF = 8.31 × 10^7^, RSD = 4.85%) and efficient catalytic reduction of organic dyes under ambient conditions (*k*_MB_ = 0.111 min^−1^, *k*_RhB_ = 0.071 min^−1^; mixed system *k*_MB_ = 0.085 min^−1^, *k*_RhB_ = 0.068 min^−1^). These performances are consistent with synergistic plasmonic-electronic effects, in which the glucose coating promotes uniform adsorption and interfacial electron transfer within a Langmuir–Hinshelwood-type surface-mediated framework, while simultaneously preventing nanoparticle aggregation. The dynamic Ag^0^/Ag^+^ redox cycle, may supported by localized surface plasmon excitation, further sustains catalytic activity. Overall, coupling green microplasma synthesis with biomolecular stabilization yields a robust dual-function hybrid material capable of ultrasensitive molecular sensing and sustainable pollutant remediation. Future work will focus on tailoring nanoparticle morphology to maximize hotspot density assessing long-term stability in complex real-world matrices, and extending this platform to multifunctional photocatalytic and environmental monitoring applications.

## Author contributions

Pham The Tan: conceptualization, supervision, writing – original draft, funding acquisition. Resources, Truong Quang Giang: data curation, methodology, investigation, Tran Thu Trang: methodology, investigation, editing, Vu Xuan Hoa: review & editing, Luu Tuan Duong: methodology, investigation, Ngo Thi Lan: review & editing, Nguyen Thi Luyen: methodology, investigation, Nguyen Van Hao: conceptualization, formal analysis, writing – review & editing.

## Conflicts of interest

The authors declare no possible conflict of interests.

## Supplementary Material

RA-016-D5RA09286H-s001

## Data Availability

The authors confirm that the data supporting the findings of this study are available within the articles. Raw data that support the findings of this study are available from the corresponding author, upon reasonable request. Supplementary information (SI): additional experimental details, characterization data, and supporting figures. See DOI: https://doi.org/10.1039/d5ra09286h.
