# Supplementary material for: Microplasma-assisted green synthesis of glucose-stabilized silver nanoparticles: a dual-functional platform for SERS detection and synergistic reduction of binary dyes
Source: RSC Adv. 2026 Jan 15;16(4):3296–313. doi: 10.1039/d5ra09286h (PMC12805421; doi:10.1039/d5ra09286h)
Supplement: RA-016-D5RA09286H-s001 [file RA-016-D5RA09286H-s001.pdf]

Supplementary Information (SI) for RSC Advances.
This journal is © The Royal Society of Chemistry 2026

Microplasma-assisted green synthesis of glucose-stabilized silver nanoparticles: a dual-functional platform for SERS detection and synergistic reduction of binary dyes

Pham The Tan[1], Truong Quang Giang[2], Tran Thu Trang[2], Vu Xuan Hoa[2], Luu Tuan Duong[2], Ngo Thi Lan[2], Nguyen Thi Luyen[2] and Nguyen Van Hao[2,*]

[1] *Hung Yen University of Technology and Education, Viet Tien ward., Hung Yen Province, Vietnam*

[2] *TNU – University of Sciences, Phan Dinh Phung Ward, Thai Nguyen Province, Vietnam*

[*] Email: haonv@tnus.edu.vn (N.V. Hao)

Prof. Dr. Nguyen Van Hao (N. V. Hao)

Institute of Science and Technology, TNU-University of Sciences

Phan Dinh Phung ward, Thai Nguyen Province, Vietnam

Email: haonv@tnus.edu.vn

S1. Synthesis of G-AgNPs

The microplasma-assisted synthesis setup for glucose-stabilized silver nanoparticles (G–AgNPs) is schematically shown in Figure S1a. A direct-current high-voltage power supply (≈2.9 kV) was used to generate an atmospheric-pressure argon microplasma in a plasma–liquid configuration. A platinum rod served as the anode, while a stainless-steel microelectrode housed in a quartz capillary acted as the cathode. The cathode tip was positioned approximately 2 mm above the liquid surface, and high-purity argon (99.99%) was supplied through the cathode at a flow rate of 50 sccm to sustain a stable discharge.

For each synthesis, 30 mL of an aqueous precursor solution containing $AgNO_3$ (0.1 – 2.0 mM) and D-glucose (10 mM) was placed in a glass beaker and subjected to ultrasonic agitation (40 kHz, 60 W) to ensure homogeneous mixing. Upon plasma ignition, a visible microplasma plume formed at the gas–liquid interface, accompanied by a gradual color change of the solution from colorless to pale yellow, indicating the formation of Ag nanoparticles (Figure S1b).

Plasma treatment was maintained for 10 min under ambient conditions to achieve complete reduction of Ag^+ ions. In selected experiments, the discharge duration was varied from 1 to 10 min at a fixed $AgNO_3$ concentration (2.0 mM) to examine time-dependent nanoparticle formation. During microplasma operation, reactive species such as solvated electrons (e^-), hydrogen radicals (H•), and hydroxyl radicals (OH•) were generated at the plasma–liquid interface, which contributed to the reduction of Ag^+ to metallic Ag^0. Simultaneously, glucose molecules acted as surface-stabilizing agents through coordination of hydroxyl groups with the nanoparticle surface, suppressing aggregation and promoting colloidal stability..

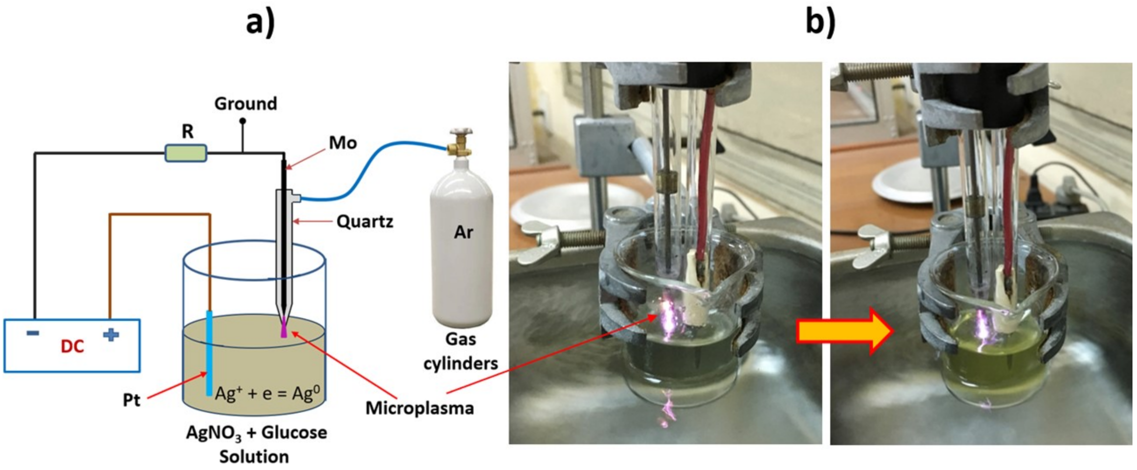

Figure S1. Schematic illustration of the plasma-liquid interaction setup for the synthesis of G-AgNPs

S2. Characterization Techniques

Transmission electron microscopy (TEM) images were acquired using a JEOL JEM-1010 microscope operated at 80 kV. Particle size distributions were obtained by measuring at least 200 nanoparticles using ImageJ software.

UV–Vis absorption spectra were recorded on a Jasco-V770 spectrophotometer in the range of 200 – 800 nm using quartz cuvettes (1 cm path length).

Fourier-transform infrared (FTIR) spectra were collected on a Jasco-4600 spectrometer in the range of 4000 – 500 cm^{-1} using KBr pellets.

X-ray diffraction (XRD) patterns were recorded on a Rigaku D2 Phaser diffractometer using Cu Kα radiation (λ = 1.5406 Å), operating at 40 kV and 40 mA, with a scan rate of 0.02° s^{-1}.

S3. SERS Measurement Details

Rhodamine 6G (Rh6G) stock solutions were prepared in deionized water and diluted to concentrations ranging from 10^{-9} to 10^{-5} M. For each measurement, 200 µL of Rh6G solution was mixed with 200 µL of G–AgNP colloid and equilibrated for 10 min prior to spectral acquisition.

Raman spectra were collected using a Horiba LabRAM HR Evolution spectrometer equipped with a 532 nm laser. The laser power was set to 1.0 mW at the sample, with a spot size of ~1 µm, an integration time of 10 s, and three accumulations per spectrum. At least nine random spots were measured for each sample to evaluate signal uniformity. Baseline correction was performed using LabSpec 6 software.

S4. Catalytic Degradation Experiments

Catalytic reduction experiments were conducted using methylene blue (MB) and rhodamine B (RhB) as model dyes. In a typical experiment, 2.5 mL of dye solution (25 mg·L^{-1}) was mixed with 0.5 mL of freshly prepared $NaBH_4$ solution (0.1 M), followed by addition of 0.5 mL of G–AgNP colloid under magnetic stirring at room temperature.

The reaction progress was monitored by recording UV–Vis absorption spectra at regular time intervals. Apparent rate constants were determined assuming pseudo-first-order kinetics with respect to dye concentration.

S5. Optical emission spectroscopy of the microplasma

Optical emission spectroscopy (OES) was conducted to identify reactive species generated during atmospheric-pressure microplasma synthesis of G–AgNPs (Figure S4). The spectrum exhibits intense Ar I emission lines in the 680 – 900 nm range (4p → 4s transitions), together

with characteristic emissions from OH radicals (308 nm), N_2 molecules (337.1 nm), atomic hydrogen (H_α, 656.3 nm), and atomic oxygen (777.4 and 844.6 nm), indicating active plasma–liquid and plasma–air interactions.

The observed N_2^+ emission at 391 nm suggests Penning ionization and charge-transfer processes induced by metastable argon species. Representative plasma reactions responsible for the formation of reactive species are summarized in Equations (S1) – (S9). Detailed emission lines and corresponding electronic transitions are listed in Table S1..

$Ar^* + N_2 \rightarrow Ar + N_2^+ + e^-$ (penning ionization) (S1)

$Ar^+ + N_2 \rightarrow Ar + N_2^+$ (charge transfer processes) (S2)

$e^- + N_2^+ \rightarrow N_2^{*+} + e^-$ (direct electron excitation) (S3)

O_2 ($N_2^{*+} + O_2 \rightarrow N_2 + O + O$ (subsequent reactions) (S4)

$Ar + e^- (E > 15.5\ eV) \rightarrow Ar^+$ (S5)

$Ar^+ + e^- (E > 19.5\ eV) \rightarrow Ar^{*+}$, (S6)

$H_2O^+ + e^- \rightarrow OH\ (A^2\Sigma^+) + H + e^-$ (S7)

$H_2O^+ + e^- \rightarrow OH\ (A^2\Sigma^+) + H$ (S8)

$Ar^* + H_2O \rightarrow OH\ (A^2\Sigma^+) + Ar + H$ (S9)

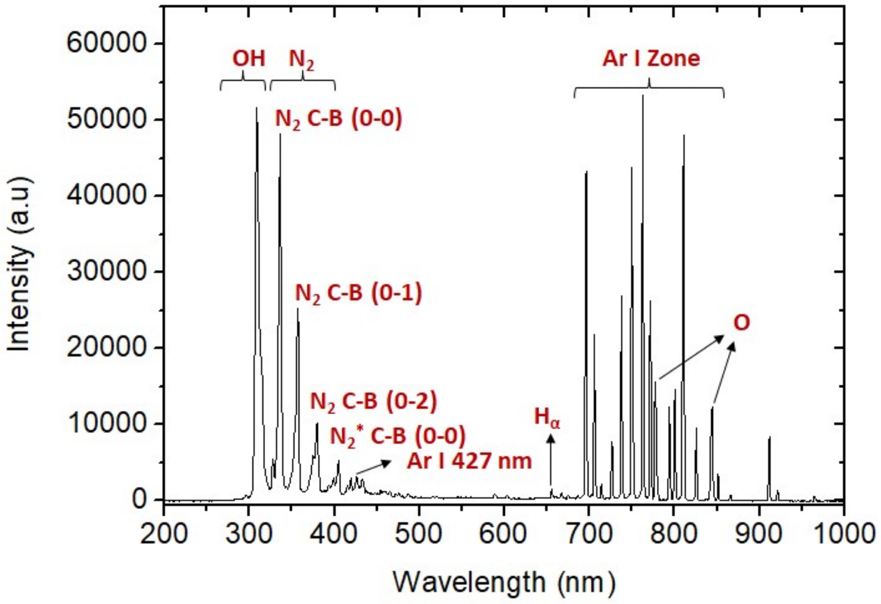

Figure S2. Optical emission spectrum of microplasma during G-AgNP synthesis

Table S1. Emission lines observed in the microplasma OES under Ar atmosphere

**Species**	**Wavelength (nm)**	**Transition**
Ar I	696,54	$3s^23p^5(^2P^0{}_{3/2})4s$ - $3s^23p^5(^2P^0{}_{1/2})4p$
Ar I	751,46	$3s^23p^5(^2P^0{}_{3/2})4s$ - $3s^23p^5(^2P^0{}_{1/2})4p$
Ar I	763,51	$3s^23p^5(^2P^0{}_{3/2})4s$ - $3s^23p^5(^2P^0{}_{1/2})4p$
Ar I	772,42	$3s^23p^5(^2P^0{}_{3/2})4s$ - $3s^23p^5(^2P^0{}_{1/2})4p$
Ar I	801,47	$3s^23p^5(^2P^0{}_{3/2})4s$ - $3s^23p^5(^2P^0{}_{1/2})4p$
Ar I	811,47	$3s^23p^5(^2P^0{}_{3/2})4s$ - $3s^23p^5(^2P^0{}_{1/2})4p$
Ar I	826,45	$3s^23p^5(^2P^0{}_{3/2})4s$ - $3s^23p^5(^2P^0{}_{1/2})4p$
Ar I	842,46	$3s^23p^5(^2P^0{}_{3/2})4s$ - $3s^23p^5(^2P^0{}_{1/2})4p$
O	777,41	$2s^22p^3(^4S^0{}_{3/2})3s$ - $2s^22p^3(^4S^0)3p$
O	844,63	$2s^22p^3(^4S^0{}_{3/2})3s$ - $2s^22p^3(^4S^0)3p$
H_α	656,28	2p – 3d
N_2	337,1	$(C\ ^3\Pi_u - B\ ^3\Pi_g)$
OH	308	$A^2\Sigma^+ \rightarrow X\ ^2\Pi$, $\Delta\nu = 0$

S6. Additional SERS Enhancement Factor Analysis

Enhancement factors (EFs) for selected Rh6G vibrational modes were calculated using the standard expression EF = $(I_{SERS}/I_{nor}) \times (C_{nor}/C_{SERS})$. The detailed EF values at $C_{SERS} = 10^{-6}$ M and 10^{-9} M are summarized in Table S2.

These data provide mode-specific insight into the SERS enhancement behavior of G–AgNP substrates and support the averaged EF values discussed in the main text.

Table S2. Enhancement factors (EF) for selected Rh6G vibrational modes on G-AgNPs substrate at $C_{SERS} = 10^{-6}$ M and 10^{-9} M ($C_{nor} = 10^{-2}$ M, $I_{nor} = 328$ a.u.).

Peak Position (cm^{-1})	Assignment (Vibrational Mode)	I_{SERS} (a.u.) at 10^{-6} M	I_{SERS} (a.u.) at 10^{-9} M	EF ($\times 10^5$) at 10^{-6} M	EF ($\times 10^7$) at 10^{-9} M
609	C-C-C ring in-plane bending	11765.2	1126.7	3.59	3.43
771	Out-of-plane C-H bending	11485.8	1025.2	3.50	3.13
1184	In-plane C-C stretching	14307.1	1290.9	4.36	3.93
1308	In-plane C-C stretching	18980.4	2137.2	5.79	6.51
1360	Aromatic C-C stretching	27472.7	2725.1	8.38	8.31
1509	In-plane C-C stretching	13124.3	1435.1	4.00	4.38

S7. Zeta Potential Analysis

Zeta potential measurements were performed to examine surface charge variations of glucose-capped silver nanoparticles (G-AgNPs) under different catalytic conditions. Pristine G-AgNPs exhibit a slightly negative zeta potential (–10.2 mV) (Fig. S5), attributable to glucose-derived surface functional groups. Upon addition of methylene blue (MB), the zeta potential changes slightly to –12.2 mV, indicating adsorption of the cationic dye onto the nanoparticle surface without complete charge neutralization. In contrast, the presence of $NaBH_4$ results in a pronounced shift to a highly negative zeta potential (–45.9 mV), suggesting strong adsorption of borohydride species and the formation of an electron-rich AgNP surface. These observations support the involvement of surface-mediated interactions between the catalyst and both reactants, consistent with a Langmuir–Hinshelwood-type catalytic pathway.

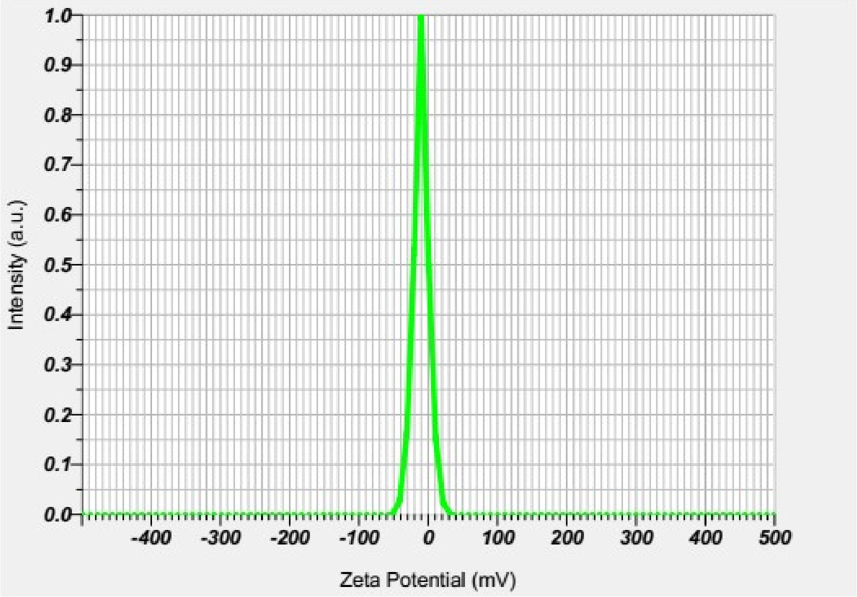

(a)

Calculation Results

Peak No.	Zeta Potential	Electrophoretic Mobility
1	-10.2 mV	-0.000079 cm2/Vs
2	--- mV	--- cm2/Vs
3	--- mV	--- cm2/Vs

Zeta Potential (Mean) : -10.2 mV
Electrophoretic Mobility Mean : -0.000079 cm2/Vs

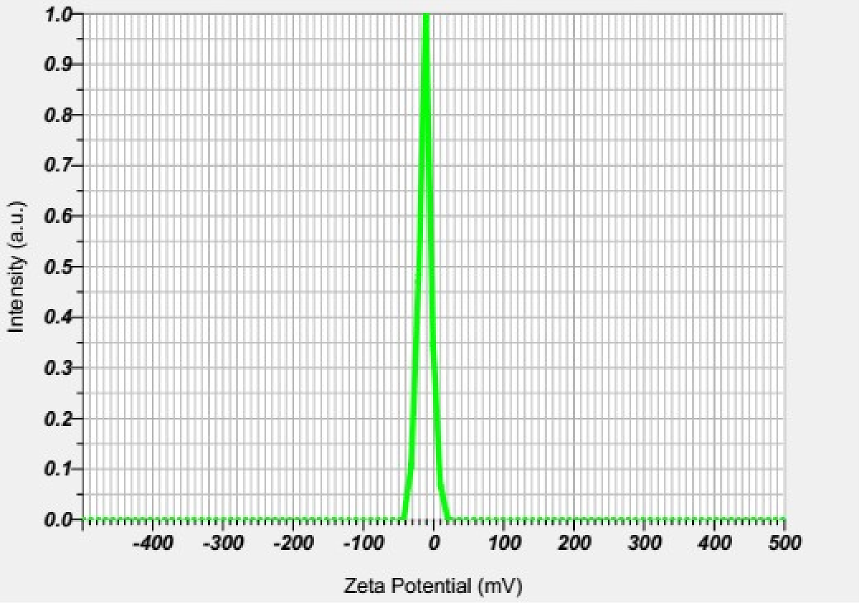

(b)

Calculation Results

Peak No.	Zeta Potential	Electrophoretic Mobility
1	-12.2 mV	-0.000094 cm2/Vs
2	--- mV	--- cm2/Vs
3	--- mV	--- cm2/Vs

Zeta Potential (Mean) : -12.2 mV
Electrophoretic Mobility Mean : -0.000094 cm2/Vs

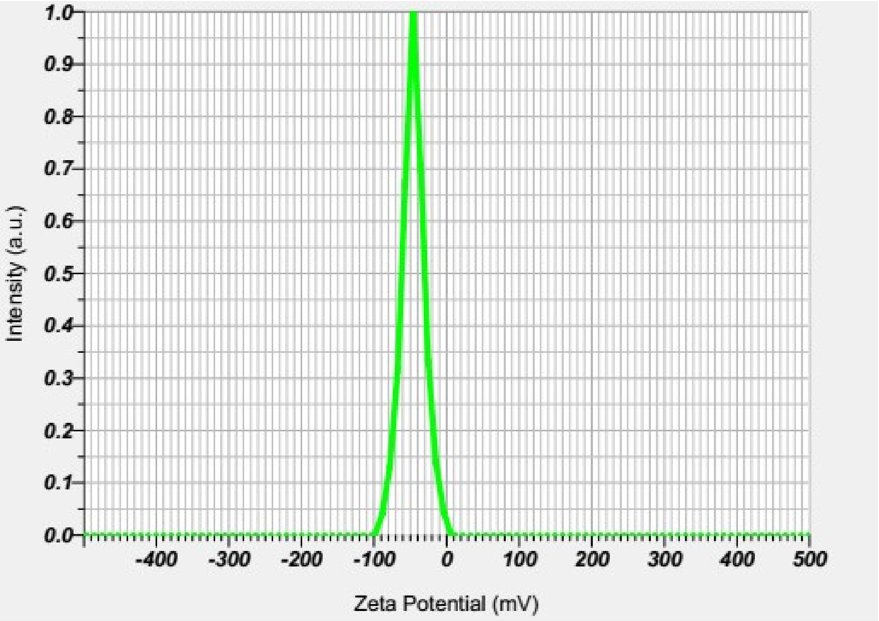

(c)

Calculation Results

Peak No.	Zeta Potential	Electrophoretic Mobility
1	-45.9 mV	-0.000355 cm2/Vs
2	--- mV	--- cm2/Vs
3	--- mV	--- cm2/Vs

Zeta Potential (Mean) : -45.9 mV
Electrophoretic Mobility Mean : -0.000355 cm2/Vs

Figure S3. Zeta potential measurements of glucose-capped silver nanoparticles (G-AgNPs) under different reaction conditions: pristine G-AgNPs (a), G-AgNPs in the presence of methylene blue (MB) (b), and G-AgNPs in the presence of $NaBH_4$ (c). The changes in surface charge indicate adsorption of both the dye molecules and the reducing agent onto the AgNP surface.
